# Innovative Artificial Intelligence System in the Children’s Hospital in Japan

**DOI:** 10.31662/jmaj.2024-0312

**Published:** 2025-02-21

**Authors:** Akihiro Umezawa, Kazuaki Nakamura, Mureo Kasahara, Takashi Igarashi

**Affiliations:** 1 National Center for Child Health and Development, Tokyo, Japan

**Keywords:** Artificial Intelligence (AI) in healthcare, Deep learning technology, Pediatric diagnosis, Medical data security, Predictive models for genetic disorders

## Abstract

The evolution of innovative artificial intelligence (AI) systems in pediatric hospitals in Japan promises benefits for patients and healthcare providers. We actively contribute to advancements in groundbreaking medical treatments by leveraging deep learning technology and using vast medical datasets. Our team of data scientists closely collaborates with departments within the hospital. Our research themes based on deep learning are wide-ranging, including acceleration of pathological diagnosis using image data, distinguishing of bacterial species, early detection of eye diseases, and prediction of genetic disorders from physical features. Furthermore, we implement Information and Communication Technology to diagnose pediatric cancer. Moreover, we predict immune responses based on genomic data and diagnose autism by quantifying behavior and communication. Our expertise extends beyond research to provide comprehensive AI development services, including data collection, annotation, high-speed computing, utilization of machine learning frameworks, design of web services, and containerization. In addition, as active members of medical AI platform collaboration partnerships, we provide unique data and analytical technologies to facilitate the development of AI development platforms. Furthermore, we address the challenges of securing medical data in the cloud to ensure compliance with stringent confidentiality standards. We will discuss AI’s advancements in pediatric hospitals and their challenges.

The advancement of cutting-edge artificial intelligence (AI) technologies in National Center for Child Health and Development holds substantial promise for enhancing patient care and supporting healthcare professionals through the Japanese Cross-Ministerial Strategic Innovation Promotion Program “Innovative AI Hospital System” ^(1)^. Through our collaboration with your esteemed pediatric institution, we are at the forefront of pioneering medical innovations by harnessing deep learning methodologies and extensive medical datasets (Figure 1). Our team of expert data scientists works synergistically with hospital departments to drive research in diverse areas influenced by deep learning. We use imaging data to expedite pathological diagnostics, differentiate bacterial species, and enable early detection of ocular conditions. Moreover, we use predictive models to identify genetic disorders from physical attributes. In addition, we leverage Information and Communication Technology to address autism spectrum disorders and pediatric oncology. Within pediatric AI hospitals, we have conducted demonstrations to refine the selection of pertinent data from imaging devices and integrate pathological and clinical information to enhance patient, family, and medical staff support. Our research also involves the prediction of immune responses through genomic data analysis and diagnosis of autism through behavioral and communicative quantification. Our expertise extends beyond research, providing a full spectrum of AI development services, including data acquisition, annotation, high-speed graphics processing unit (GPU) computing, machine learning framework application, web service design, and containerization. As active contributors to medical AI platform collaborations, we provide unique data and analytical technologies to advance AI platform development. Furthermore, we address the critical issue of securing medical data in cloud environments to ensure adherence to rigorous confidentiality standards. We will delve into AI’s progress in pediatric hospitals and discuss the associated challenges.

**Figure 1.**
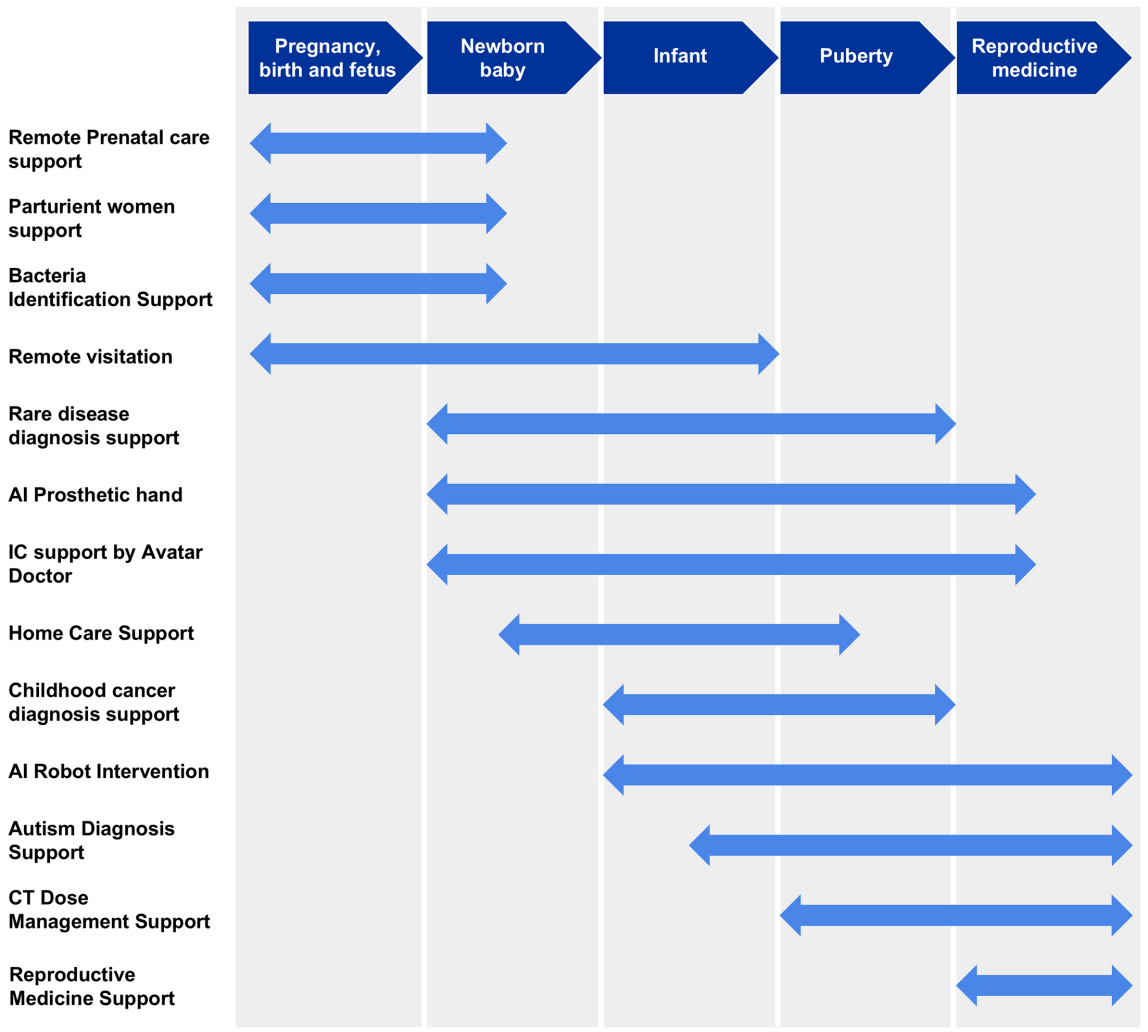
AI hospital project at NCCHD NCCHD has been advancing the development and social implementation of an advanced diagnostic and treatment system based on an AI hospital under the Cross-Ministerial Strategic Innovation Promotion Program, a Japanese national project. We have been developing AI systems across a broad spectrum, from pregnancy and childbirth to infancy and adolescence, and are extending them into adult medicine, including reproductive medicine. AI, artificial intelligence; IC, informed consent; CT, computed tomography.

## AI in Obstetrics: Development of Diagnostic Support and Assistance Systems

**Figure 2.**
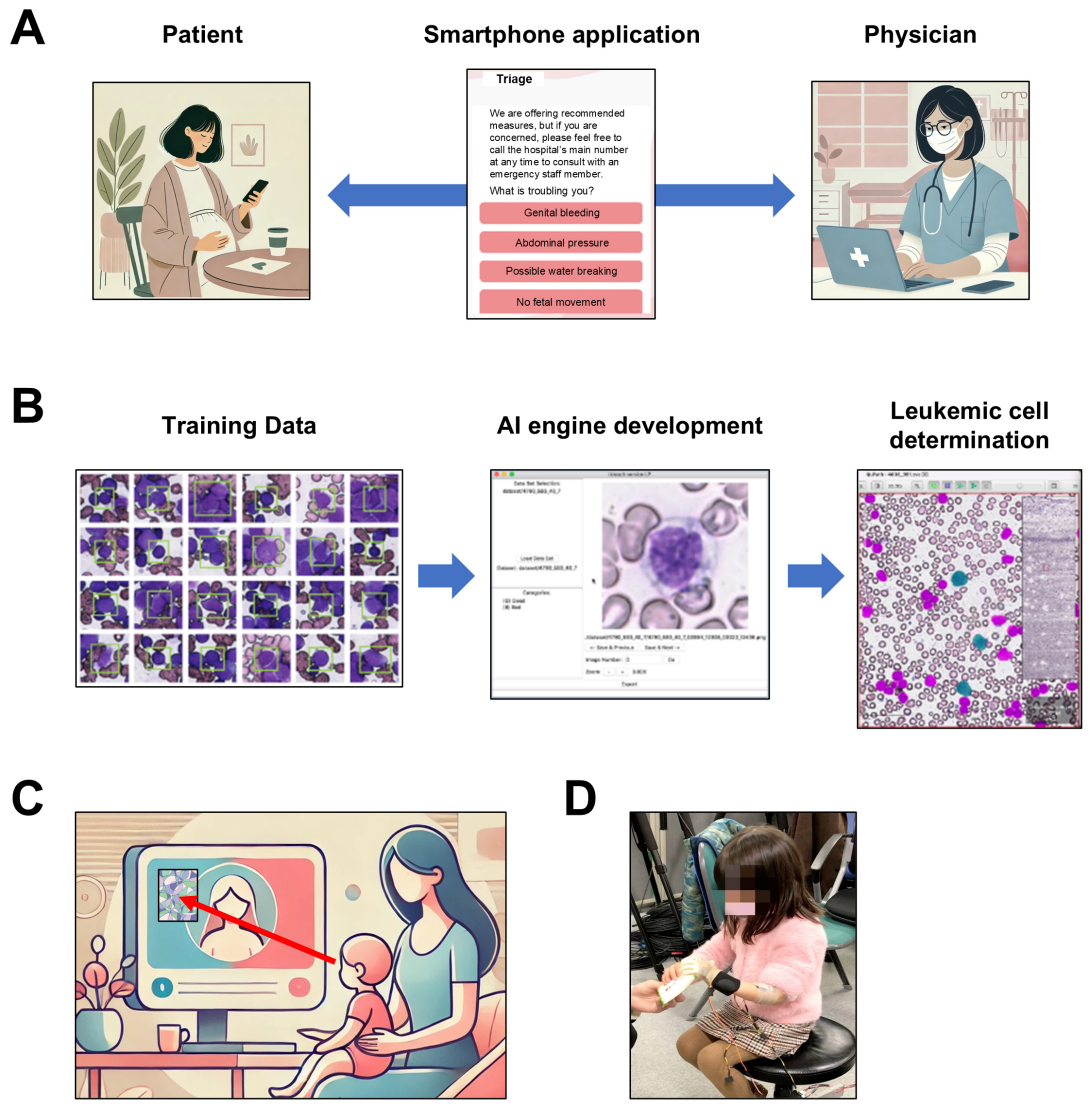
Development of AI-based systems for children’s and women’s health and medicine A. AI in Obstetrics The first step is to develop an AI-based prenatal checkup support system. All pregnant women who visit our hospital will be asked to download a remote prenatal checkup system application. With this application, pregnant women can make appointments for remote prenatal checkups. Doctors can review symptoms and other information pregnant women enter in advance, enabling remote prenatal checkups via PC or smartphone. This system has been exceptionally well received by pregnant women with children, for whom a hospital visit is inconvenient, as well as by pregnant women with busy work schedules who find it challenging to take time off, and those who wish to have a checkup during their workday without visiting the hospital. This system is expected to be used for remote prenatal care in underserved or remote areas that lack obstetricians. In addition, we are developing a triage system where pregnant women can input their concerns and receive AI-based recommendations. The AI assesses the urgency of the situation, advising whether to contact a hospital or monitor the situation from home. We plan to implement the triage system as an additional feature in the remote prenatal checkup system. B. Diagnostic support system for pediatric cancer I want to shift the focus from obstetrics to pediatric cancer. We have developed an AI diagnostic support system for pediatric leukemia bone marrow smears. Diagnosing leukemia is a complex and time-consuming task for specialists analyzing blood cells under a microscope. Our AI, trained on 100,000 annotated cell images, can detect blasts (abnormal cells) in a bone marrow smear with an accuracy of 94%. The AI can analyze 100 cells in approximately 10 s. This system provides objective, reliable, and efficient diagnostic support for childhood leukemia. We plan to conduct international clinical trials and develop the system as a certified medical device. C. Eye gaze finder We are developing an AI-based system to support the diagnosis of Autism Spectrum Disorder (ASD) using eye-tracking. Previous studies reported that children with ASD prefer geometric patterns over human faces. Using eye gaze measurements in 2-year-olds, our AI achieved 81% sensitivity and 85% specificity in ASD diagnosis. This system can identify ASD in 2-year-olds who are otherwise difficult to diagnose. Children identified by this system at age 2 were confirmed to have ASD by specialists at age 5. Thus, this system enables early diagnosis and intervention, potentially mitigating ASD symptoms. D. AI-assisted prosthetic hand We are developing an AI-assisted prosthetic hand for children facing difficulties using traditional myoelectric prostheses. Conventional myoelectric devices often require extensive rehabilitation, which can be cumbersome and consistently unsuccessful. Our AI prosthesis adapts to the patient’s unique muscle signals, enabling more intuitive and immediate prosthetic hand control. Informed consent for publication in the trial was obtained after thoroughly explaining the study to the surrogate (parents or legal guardians). This figure is reproduced from Takagi T., 'Figure 2C. A Case of Congenital Hand Deformity,' A New Hand: The Present and Future of Prosthetic Hands, published in Orthopedics and Traumatology, Vol. 67, No. 12, 2024, pp. 1211–1217, with permission from Kanehara & Co., Ltd.

The COVID-19 pandemic posed unprecedented challenges to healthcare systems worldwide, forcing rapid adaptations in delivering care. Pregnant women were particularly affected, with heightened concerns regarding the risk of infection and restricted access to in-person care. In response to these challenges, NCCHD conducted a pilot study on an innovative smartphone app-based pregnancy care support and triage system (Figure 2A). This system enabled contactless medical care and was highly appreciated by pregnant women, particularly those managing pregnancy and work or caring for children. The development of this pregnancy care system represented a considerable step in ensuring that pregnant women could continue receiving necessary medical care while minimizing exposure to the virus. This smartphone app-based system enabled pregnant women to engage in virtual consultations with healthcare providers from the safety of their homes.

One of the most critical outcomes of this system is the triage function. The application incorporates a triage system that helps pregnant women evaluate their symptoms and determine whether immediate medical attention is needed or if the issue can be remotely managed. This feature substantially alleviates the anxiety of pregnant women, particularly when experiencing common symptoms during pregnancy. The application provides customized recommendations based on the woman’s medical history and current symptoms, providing reassurance. Furthermore, this triage system enables physicians to focus on patients requiring urgent care while efficiently managing routine check-ins and consultations via the application.

The application mainly benefits pregnant women caring for children or balancing work and pregnancy. These women often face the added pressure of ensuring proper prenatal care while managing household and work responsibilities. The convenience of this application, which enables them to receive medical advice and support from home, has been transformative. Through the application, they can maintain consistent, high-quality care during pregnancy while fulfilling their responsibilities. Feedback from users, positive and negative, reflects the impact of this telemedicine initiative. The success of this system also highlights the potential for remote pregnancy care beyond the pandemic. As healthcare systems worldwide continue to adopt digital health solutions, the lessons learned from the model of NCCHD may influence the development of similar systems in other regions.

## Development of an AI-based Diagnostic Support System for Pediatric Cancer

AI-driven precision medicine is making significant strides in oncology ^(2), (3)^. Motohiro Kato’s team has developed an AI diagnostic support system for pediatric leukemia, which emphasizes analyzing bone marrow smears (Figure 2B). Diagnosing leukemia is a complex and time-consuming task for specialists who must analyze blood cells under a microscope. Our AI engine, trained on 100,000 annotated cell images, excels in detecting blasts, i.e., abnormal cells, in bone marrow smears with an accuracy of 94%. The AI can analyze 100 cells in approximately 10 s, accelerating the diagnostic process. This system provides objective, reliable, and efficient diagnostic support for childhood leukemia, surpassing traditional methods employed by pediatric hematologists.

One of the key advantages of this AI system is its consistent and objective analysis, which eliminates the subjectivity and variability inherent in the manual inspection of blood smears. An important innovation of this system is its capacity to calculate the “percentage of leukemia remaining” in blood smears during treatment, providing a detailed and accurate assessment of treatment efficacy. Kato’s team plans to conduct international clinical trials and develop the system as a certified medical device, with the aim of enhancing the accuracy and efficiency of leukemia diagnostics worldwide. This advancement represents an important step in pediatrics, providing clinicians with better tools for treatment adjustment and patient management, in addition to previous approaches ^(4), (5)^.

Furthermore, a leukemia immunodiagnostic system and a genome report drafting system are being developed. The leukemia immunodiagnostic system has been completed and is ready for external presentation and collaboration with partner companies. The existing data utilized for system construction for the genome report drafting system have been organized, and development is currently in progress. Clinical trials for testing the system are planned to begin.

## Advanced Diagnostic Support System for Autism Spectrum Disorder Using Eye-tracking

An advanced AI-based system is currently developed to support the early diagnosis of Autism Spectrum Disorder (ASD). This innovative system leverages eye-tracking technology to monitor gaze patterns in young children and identify atypical eye movements associated with ASD (Figure 2C). The main objective is to promote early detection and enable timely intervention, ultimately leading to improved long-term outcomes. Eye-tracking is noninvasive and provides objective insights into how children visually interact with their surroundings, thereby providing valuable information on social and cognitive development. By using AI to analyze gaze patterns, the system serves as a powerful tool for healthcare professionals to detect ASD at an early stage. The system is designed for integration into routine developmental screenings, such as those conducted at 18 months and 3 years of age. During these screenings, the AI-driven eye-tracking system monitors the child’s gaze and attention, determining whether their visual patterns align with typical developmental milestones or indicate signs of ASD. This approach facilitates early identification and offers objective, quantitative measures that support accurate diagnoses.

Children at risk for ASD can be referred to specialized medical facilities for further evaluation. At this stage, the AI system can be used again to reinforce initial findings and assist with more detailed assessments. This multistep process enhances the reliability of the diagnosis by complementing early detection with additional data. The system reduces variability in traditional diagnostic methods by providing consistent, objective standards and improving accuracy. One of the most significant advantages of the system is its ability to confirm the effectiveness of early interventions. Children diagnosed with ASD at age 2 using this technology are reassessed at age 5, with their developmental progress closely monitored. Follow-up evaluations have yielded promising results, suggesting that early detection and timely intervention significantly improve social, cognitive, and behavioral outcomes. Early diagnosis opens the door to more effective treatments, such as behavioral therapy, speech therapy, and social skills training. These are particularly beneficial when introduced during the critical early years of development.

## AI-assisted Prosthetic Hand for Children

The AI-assisted prosthetic hand offers a breakthrough solution for children struggling with conventional myoelectric prostheses (Figure 2D). Unlike traditional devices, this innovative prosthetic adapts to a child’s unique muscle signals, substantially reducing the rehabilitation period and improving functionality from the first day of use. The AI system learns each patient’s specific muscle tone and signal patterns before use. This calibration enables the prosthetic to accurately respond to subtle muscle movements, providing a more intuitive and responsive experience. After calibration, the child’s arm muscles are connected to the prosthetic hand, which uses AI to interpret muscle signals in real time. Consequently, children can quickly grasp and release objects and perform hand movements almost immediately, enhancing their independence and quality of life.

One of the key advantages of this AI-assisted prosthetic is its ability to drastically shorten the rehabilitation period. Traditional prostheses require extensive training, often taking weeks or months for children to adapt to the device. Contrarily, this AI-powered prosthetic enables near-instant use, with children performing daily activities within hours of the first trial. This rapid adaptation helps build confidence and reduces the emotional strain of learning to use a prosthesis. Designed with comfort and adaptability, the lightweight prosthetic can adjust as the child grows, making it suitable for long-term use without frequent replacements. The adaptability of the system ensures that the prosthesis remains responsive to the child’s changing muscle signals.

## Development of an AI-based Bacterial Identification Support System

**Figure 3.**
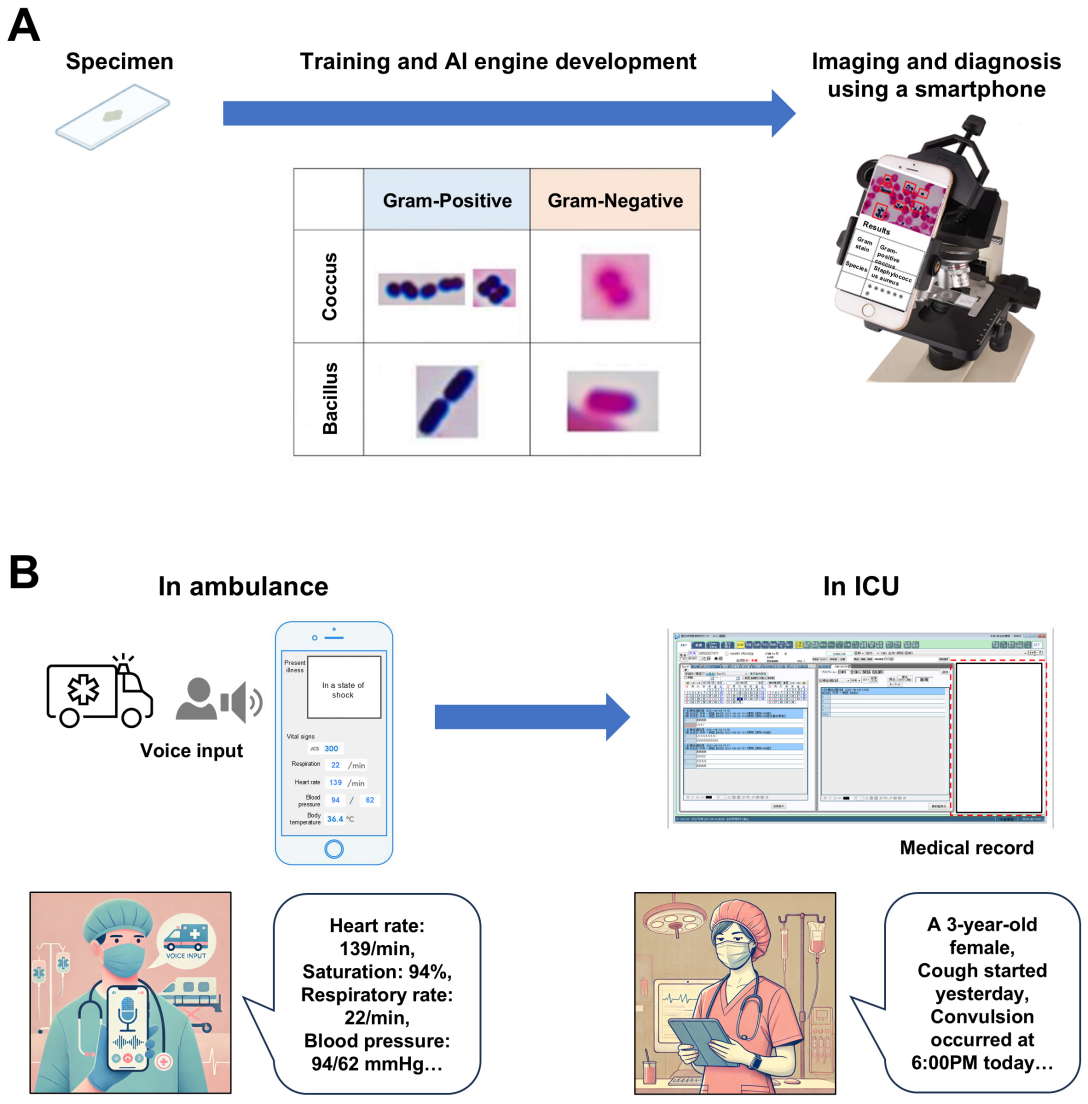
AI system for bacterial identification and pediatric emergency medicine A. Bacterial identification support system NCCHD developed an AI system to diagnose bacterial species from blood cultures. Using Gram staining―a standard test that differentiates bacteria by their morphology and color under a microscope―our AI achieved an accuracy of 97% in Gram stain classification and 88% in bacterial species identification. This system can be remotely used by attaching a smartphone to the microscope via a specialized attachment. B. Automated medical record system using the AI Voice technology Voice input is automatically captured on a smartphone or tablet and displayed on the screen as text. This voice record can then be transferred to the EMR via a QR code, automatically integrated into the database. This system facilitates accurate and timely record-keeping, particularly in high-pressure environments, such as ambulances and intensive care units. These corrections maintain the scientific and professional tone of the original text while ensuring clarity and accuracy.

An innovative AI-based system has been developed to enhance the rapid and accurate identification of bacteria using Gram staining (Figure 3A). This cutting-edge technology achieves an impressive accuracy of 97% in Gram stain classification and 88% in bacterial identification. The revolutionization of traditional microscopy methods yields swift and precise diagnostic results, substantially improving the efficiency of bacterial identification. The AI-based system is being collaboratively evaluated to ensure its effectiveness as a medical device. This extensive evaluation process assesses the usability and accuracy of the system in various settings ^(6), (7)^. The AI system is also integrated into the lab infrastructure, facilitating its validation in different locations ^(8), (9)^. This approach confirms the system’s performance under real-world conditions and allows continuous improvement. The collected images from these evaluations are used for AI retraining, enhancing the accuracy and reliability of the system over time ^(10), (11)^. Furthermore, discussions with potential companies interested in adopting this advanced technology are ongoing.

## Automated Medical Record System Using AI Voice Technology

NCCHD is leading the way in the integration of AI voice technology into electronic medical records (EMR) systems, specifically targeting high-pressure environments such as ambulances and ICUs (Figure 3B). This innovative system enables hands-free, real-time documentation, greatly enhancing the accuracy and efficiency of medical records. The process begins with voice input captured via a smartphone or tablet. The spoken words are instantly converted to text and displayed on the screen of the device, allowing immediate visual confirmation. This seamless voice-to-text conversion ensures that critical information is accurately recorded without manual data entry, reducing the risk of errors. Once the voice recording is made, it is transferred to the EMR system through a QR code. This integration process ensures that the recorded data is securely and efficiently incorporated into the medical record database. By automating the data entry process, this system improves the documentation accuracy and streamlines workflows in high-pressure settings. This AI-driven solution represents a significant advancement in medical record-keeping, providing real-time, hands-free documentation that supports clinicians in delivering timely and precise care.

## Utilization of Electronic Medical Record Systems in Next-generation Smart Hospitals

In the construction of next-generation smart hospitals, a sophisticated EMR system is being created using HL7 FHIR servers. This system efficiently summarizes extensive data registered in EMRs, generating drafts of medical documents based on specific needs. By leveraging the HL7 FHIR standard, this system can be implemented independently of the EMR vendors, making it versatile and adaptable across platforms. The core functionality of this system lies in its ability to filter and extract relevant information from vast amounts of medical data, enabling the streamlined creation of medical document drafts. Previously, the creation of a referral document manually took approximately 30 min. However, with the new system, substantial time savings are achieved as users only need to adjust the draft document rather than starting from scratch.

Implementing the Dynamic Case Summary system of NCCHD involves upgrading EMRs to include a launch button for easy access. Initial testing with a few physicians from selected departments has been completed. The Dynamic Case Summary system structures and digitizes EMR data into the internationally recognized HL7 FHIR format, enabling the dynamic extraction of relevant medical information from extensive records. The system is designed for easy navigation, making it possible to generate accurate drafts of medical documents. The goal is to support various types of medical documents. Although generative AI has not yet been employed, its integration could enhance the naturalness of the generated medical documents in the future. In addition, with the Ministry of Health, Labour and Welfare of Japan, indicating plans for an EMR information-sharing service, there is potential for the Dynamic Case Summary system to facilitate digital data-sharing between medical institutions. This would allow documents created by the system to be shared across different facilities, aligning with future expectations for digital interoperability in healthcare.

## Conclusion

The AI Hospital project in Japan began with the ambitious goal of providing an accessible high-quality medical care nationwide. This initiative aims to enhance the well-being of healthcare providers while ensuring compassionate care for patients. We have recognized the critical need for AI in pediatric hospitals and have made substantial strides in its implementation and validation. Noteworthy advancements include AI-powered robotic dogs and sensors that measure breathing and heart rate. In addition, we have used AI to address challenges in pediatric medicine, such as employing AI for cancer pathology diagnosis and identifying bacteria responsible for sepsis. By feeding images into AI systems, they can identify bacterial species at a level comparable to expert analysis in just 10 s. This capability enables rapid and precise diagnoses, substantially improving patient outcomes. However, as AI advances in pediatric hospitals, it faces several challenges. These include technical integration, cost considerations, and ethical concerns surrounding data security and patient privacy. As we discuss the progress of AI in pediatric care and the hurdles it encounters, ongoing efforts are warranted to realize its potential and fully overcome existing obstacles.

## Article Information

### Conflicts of Interest

None

### Sources of Funding

This work was supported by Council for Science, Technology and Innovation (CSTI), Cross-ministerial Strategic Innovation Promotion Program (SIP), “Innovative AI Hospital System” (Funding Agency: National Institute of Biomedical Innovation, Health and Nutrition (NIBIOHN)).

### Acknowledgement

We sincerely thank the following Principal Investigators for their invaluable contributions to this research: Drs. Koji Okamura and Toshihiro Matsui for the AI-based bacterial identification support system; Dr. Nagayoshi Umehara for the diagnostic support and assistance systems; Dr. Takehiko Takagi for the AI-assisted prosthetic hand for children; Dr. Tatsuya Koeda for the advanced diagnostic support system for ASD; Dr. Motohiro Kato for the AI-based diagnostic support system for pediatric cancer; and Dr. Satoko Uematsu for the automated medical record system utilizing the AI voice technology.

### Author Contributions

AU is responsible for the content of this paper; KN is the project management leader; MK is the hospital director in charge of the AI Hospital; TI is the overall director of the AI Hospital project.

### Disclaimer

Takashi Igarashi is the Deputy Editor of JMA Journal and on the journal’s Editorial Staff. He was not involved in the editorial evaluation or decision to accept this article for publication at all.
